# Hyperinsulinemia Promotes Esophageal Cancer Development in a Surgically-Induced Duodeno-Esophageal Reflux Murine Model

**DOI:** 10.3390/ijms19041198

**Published:** 2018-04-14

**Authors:** Diletta Arcidiacono, Arben Dedja, Cinzia Giacometti, Matteo Fassan, Daniele Nucci, Simona Francia, Federico Fabris, Alice Zaramella, Emily J. Gallagher, Mauro Cassaro, Massimo Rugge, Derek LeRoith, Alfredo Alberti, Stefano Realdon

**Affiliations:** 1 Digestive Endoscopy Unit, Veneto Institute of Oncology IOV-IRCCS, via Gattamelata, 64, 35128 Padua, Italy; daniele.nucci@iov.veneto.it (D.N.); stefano.realdon@iov.veneto.it (S.R.); 2 Department of Cardiac, Thoracic and Vascular Sciences, University of Padua, via Giustiniani 2, 35128 Padua, Italy; arben.dedja.pd@gmail.com (A.D.); 3 Anatomic Pathology Unit, ULSS 6 Euganea, via Cosma, 1, Camposampiero, 35012 Padua, Italy; cinzia.giacometti@gmail.com (C.G.); mauro.cassaro@ulss15.pd.it (M.C.); 4 Department of Medicine, Surgical Pathology & Cytopathology Unit, University of Padua, via Giustiniani 2, 35128 Padua, Italy; matteo.fassan@gmail.com (M.F.); massimo.rugge@unipd.it (M.R.); 5 Venetian Institute of Molecular Medicine-VIMM, via Orus, 2, 35129 Padua, Italy; francia.simona@gmail.com (S.F.); federico.fabris.5@studenti.unipd.it (F.F.); alice.zaramella@studenti.unipd.it (A.Z.); alfredo.alberti@unipd.it (A.A.); 6 Department of Biomedical Sciences, University of Padua, via Bassi, 58/B, 35131, Padua, Italy; 7 Department of Molecular Medicine, University of Padua, via Gabelli, 63, 35128 Padua, Italy; 8 Division of Endocrinology, Icahn School of Medicine at Mount Sinai, 1 Gustave L. Levy Pl, New York, NY 10029, USA; emily.gallagher@mssm.edu (E.J.G.); derek.leroith@mssm.edu (D.L.)

**Keywords:** hyperinsulinemia, duodenal reflux, esophageal cancer, MKR mouse model, insulin-like growth factor 1 receptor, human epidermal growth factor receptor 2

## Abstract

Hyperinsulinemia could have a role in the growing incidence of esophageal adenocarcinoma (EAC) and its pre-cancerous lesion, Barrett’s Esophagus, a possible consequence of Gastro-Esophageal Reflux Disease. Obesity is known to mediate esophageal carcinogenesis through different mechanisms including insulin-resistance leading to hyperinsulinemia, which may mediate cancer progression via the insulin/insulin-like growth factor axis. We used the hyperinsulinemic non-obese FVB/N (Friend leukemia virus B strain) MKR (muscle (M)-IGF1R-lysine (K)-arginine (R) mouse model to evaluate the exclusive role of hyperinsulinemia in the pathogenesis of EAC related to duodeno-esophageal reflux. FVB/N wild-type (WT) and MKR mice underwent jejunum-esophageal anastomosis side—to end with the exclusion of the stomach. Thirty weeks after surgery, the esophagus was processed for histological, immunological and insulin/Insulin-like growth factor 1 (IGF1) signal transduction analyses. Most of the WT mice (63.1%) developed dysplasia, whereas most of the MKR mice (74.3%) developed squamous cell and adenosquamous carcinomas, both expressing Human Epidermal growth factor receptor 2 (HER2). Hyperinsulinemia significantly increased esophageal cancer incidence in the presence of duodenal-reflux. Insulin receptor (IR) and IGF1 receptor (IGF1R) were overexpressed in the hyperinsulinemic condition. IGF1R, through ERK1/2 mitogenic pattern activation, seems to be involved in cancer onset. Hyperinsulinemia-induced IGF1R and HER2 up-regulation could also increase the possibility of forming of IGF1R/HER2 heterodimers to support cell growth/proliferation/progression in esophageal carcinogenesis.

## 1. Introduction

Esophageal adenocarcinoma (EAC) is the most rapidly increasing form of cancer in some populations [1]. Barrett’s mucosa is assumed to be a pre-cancerous lesion for EAC [2] and it is defined as columnar epithelium, replacing esophageal squamous epithelium, initiated by reparative processes in the setting of gastro-esophageal reflux disease (GERD). It is reported that not only gastric acid, but also the duodenal basic content (bile acids in particular) play an important role in the generation of columnar metaplasia [3]. Obesity has been previously identified as an important risk factor for EAC, which is known to predispose patients to increased gastro-esophageal reflux, due to anatomic factors such as increased abdominal and intraperitoneal adiposity, and hiatal hernia formation [4]. Obesity is also known to mediate cancer progression through insulin-resistance, inflammation and oxidative stress, and production of fat-associated hormones (adipokines). Obesity is associated with a number of comorbidities, among them hypertension, hyperlipidemia, and diabetes mellitus [5]. Previous reports have shown that diabetes increases the risk for several types of cancer and treatment with the antidiabetic drug metformin may decrease this risk [6,7,8,9]. Moreover, hyperinsulinemia and insulin resistance may mediate cancer progression via the insulin/insulin-like growth factor axis [8,10]. The specific mechanisms involved in this tumor-promoting activity are unclear. Furthermore, Human Epidermal growth factor receptor 2 (HER2) signaling activation is known to have a pivotal role in EAC carcinogenesis and disease progression. A relationship between insulin and HER2 signaling was already demonstrated for breast cancer [11,12,13] and could also be hypothesized in this context.

The aim of this study is to evaluate the exclusive role of hyperinsulinemia in the pathogenesis of EAC related to chronic duodeno-esophageal reflux in a pre-diabetes context and in the absence of obesity as confounding factor associated with cancer risk. To this purpose we used an animal model (MKR mouse) that has already been largely used to demonstrate the specific role of hyperinsulinemia in the development of other epithelial cancers such as breast cancer [8]. MKR mice were generated by overexpressing a dominant-negative IGF1R specifically in skeletal muscle. The transgene encodes for human IGF1R; it has a point mutation in the ATP-binding domain and is driven by the muscle creatine kinase promoter, resulting in the inactivation of IR and IGF1R exclusively in skeletal muscle. Mice develop a mild diabetic phenotype, which recapitulates the early stages of type 2 diabetes (pre-diabetes) in humans. A mild dysglycemia, a marked hyperinsulinemia, and severe insulin resistance are the major metabolic abnormalities detected in MKR female mice during their whole life; the severe and uncompensated insulin resistance leads to diabetes development in old age MKR male mice. Importantly, MKR mice are not obese; they have moderately reduced total body weight and body adiposity compared with their control littermates [8].

## 2. Results

### 2.1. Macroscopic Findings in Operated Wild-Type and MKR Mice

**Figure 1:**
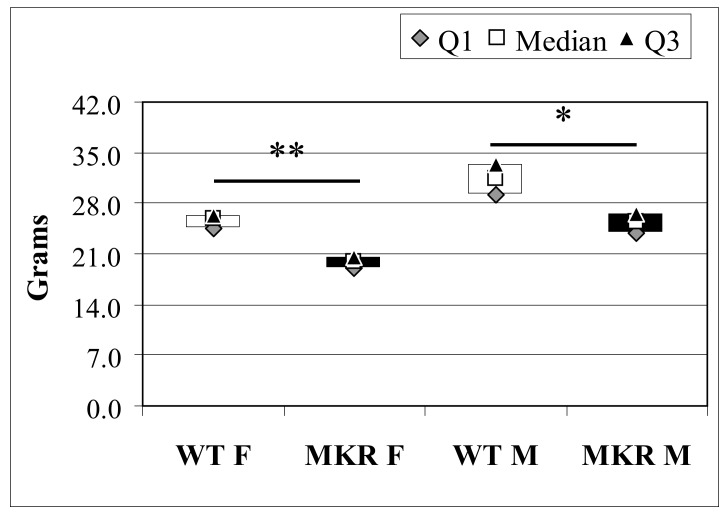
Total body weight of 13-week-old wild-type (WT) and hyperinsulinemic (MKR) mice. Data were expressed as Median (Q1; Q3). F: Female mice; M: Male mice. * Mann-Whitney *U*-test, *p* = 3.8 × 10^−4^; ** Mann-Whitney *U*-test *p* = 2.0 × 10^−6^.

Body weight was measured in all animals before surgery. According to previous work [8,14], MKR mice had reduced total body weight (BW) compared to WT controls (Figure 1). Six animals died in the post-operative period due to complications, such as malnutrition and/or breathing difficulties. A total of 73 out of 79 (92.4%) mice completed the study. At the time of euthanasia, most of the operated animals showed an enlargement of the esophageal cavity (especially the lower and middle parts), increased thickness, and epithelium hyperkeratinization. Some animals showed cardiac hypertrophy and splenomegaly (36.8% and 45.7% in WT and MKR groups, respectively; *p* = 0.482), possibly secondary to anemia. As expected, in all animals the stomach was atrophied.

### 2.2. Histological Findings in Operated Wild-Type and MKR Mice

According to histological findings, animals were grouped into four main categories: (1) No significant lesions with normal squamous epithelium; (2) Esophagitis; (3) Intraepithelial/noninvasive neoplasia, formerly known as dysplasia; (4) Cancer. Non-ulcerative and ulcerative esophagitis (category 2) was defined as sub-epithelial inflammatory infiltrate (mostly coexisting with intraepithelial leukocytes) which is often coexistent with granulation tissue and hyperplastic-regenerative changes of the surrounding epithelium. Intraepithelial/noninvasive neoplasia (category 3) was defined as the presence of cytology atypia (of low or high grade) in the absence of an architectural disarrangement or subepithelial invasion. Cancer (category 4) was defined as having invasive lesions consisting of squamous epithelial cells or of solid nests of epithelia with focal glandular differentiation (Esophageal Squamous Cell Carcinoma-ESCC). In some case, the neoplastic lesions featured both squamous and glandular differentiation (Esophageal Adeno-Squamous Carcinoma-EASC).

Histological analyses revealed that 30 weeks after surgery, both WT and MKR mice, developed esophageal neoplastic lesions.

In particular, most of the WT mice (*n* = 24 out of 38; 63.1%) developed esophageal dysplasia; carcinoma was found in 31.6% of WT mice. However, most of the MKR mice (*n* = 26 out of 35; 74.3%) developed esophageal cancer. Dysplasia was found in 11.4% of MKR mice that were subjected to duodenal reflux (MKR vs. WT: OR 0.07, 95% CI 0.02–0.26, *p* < 0.0001 for dysplasia; OR 6.26, 95% CI 2.25–17.38, *p* = 0.0004 for carcinoma). In the remaining animals, histologic features of esophagitis, including marked hyperplastic changes, such as increased thickness of the esophageal wall squamous epithelium, and hyperkeratosis, were found (11.4% of MKR mice). No esophageal lesion was found in 5.3% and 2.9% of WT and MKR mice, respectively (Table 1).

### 2.3. Lesion Histology and Gender Difference

Six animals out of 18 (33.3%) of WT males that underwent surgery developed cancer. Sixteen out of 17 (94.4%) of MKR males that underwent surgery developed cancer (OR 32.00, 95% CI 3.39–302.23, *p* = 0.002). Six animals out of 20 (30.0%) of WT females that underwent surgery developed cancer. Ten out of 18 (55.5%) of MKR females that underwent surgery developed cancer (OR 2.92, 95% CI 0.77–11.07, *p* = 0.116). EASC development was significantly associated to male gender (OR 3.56, 95% CI 1.18–10.72, *p* = 0.024) (Table 2). No differences in cancer incidence were found between WT males and WT females (OR 1.17, 95% CI 0.30–4.59, *p* = 0.825). On the contrary, cancer incidence on MKR males was higher compared to MKR females (OR 12.80, 95% CI 1.38–118.32, *p* = 0.025).

All (100%) low-grade dysplastic lesions (LGD) affected esophageal squamous epithelium and were predominantly found in WT females (Fisher’s exact test: *p* = 0.001 and *p* = 0.006 WT females vs. WT males and vs. MKR females, respectively). High-grade dysplastic lesions (HGD) were found in 21.0% of WT mice (75% males) and in 5.7% of MKR mice (100% females, no dysplastic lesions were found in MKR males) and both esophageal squamous and glandular components were affected. Table 2 summarizes histological analysis result for each animal group.

### 2.4. Basal Serum Metabolic Parameters

To understand the metabolic status of the mice before surgery, fasting blood glucose and serum insulin, C-peptide and leptin levels were measured at 13 weeks of age in non-operated animals. After two hours of fasting, glucose levels were lower in female than in male mice, without any significant difference between the four groups of mice (Kruskal-Wallis test result: *p* = 0.078). The Mann Whitney *U*-test showed that the differences between female and male MKR mice were close to statistical significance (*p* = 0.046) (Table S1, Supplementary Materials). Cuzick’s test for trend showed that glucose level increased across the four ordered groups: WT females, WT males, MKR females, MKR males (z = +2.04; *p* = 0.041). As expected, MKR mice showed marked hyperinsulinemia, 6–8-fold higher than WT mice of the same gender. Furthermore, our data showed significantly higher serum insulin in WT males compared to WT females (*p* = 1.6 × 10^−3^) (Table S1, Supplementary Materials). Within MKR group, males had higher serum insulin than females, but the difference did not reach statistical significance.

C-peptide levels, a more stable and accurate marker of endogenous insulin secretion, were higher in MKR mice compared to their WT counterparts (Table S1, Supplementary Materials). Our data showed no significant difference between males and females belonging to the same group. Cuzick trend test showed significant increased levels of both serum insulin and C-peptide across the four groups (z = +5.25, *p* < 1 × 10^−4^ and z = +4.03, *p* < 1 × 10^−4^, respectively).

Leptin serum levels were significantly lower in MKR mice compared to age- and sex-matched WT mice. No differences in leptin levels between females and males belonging to the same group were found (Table S1, Supplementary Materials), although the differences between WT males and females were close to statistical significance (*p* = 0.021). No differences in IL-6 serum levels among the four groups were found (27.1 and 24.6 in WT females and males, respectively; 23.1 and 21.7 in MKR females and males, respectively).

### 2.5. Basal Insulin Signaling in Mice Esophageal Tissue

To study the effect of hyperinsulinemia on esophageal cancer development, we evaluated insulin signal transduction in esophageal tissue of non-operated animals at 13 weeks of age. It is known that the biological effects of insulin are mediated by the structurally related Insulin receptor (IR) and insulin-like growth factor receptor 1 (IGF1R). At physiological levels, insulin binds to the IR, while IGF1 binds primarily to the IGF1R and IGF1R/IR hybrid receptors, and IGF2 binds to the IGF1R and IR isoform A. IR and IGF1R transduce an intracellular signal primarily through the PI3K pathway and the mitogen-activated protein kinase (MAPK) pathway [15]. We evaluated total protein expression and phosphorylation level of IR and IGF1R from the distal esophageal segment of mice by Luminex-xMAP^®^ technology.

IR is a glycoprotein consisting of an extracellular α-subunit and a transmembrane β-subunit. Insulin binding to the α-subunit results in the dimerization of the receptor, with the formation of the α_2_β_2_ complex in the cell membrane, and the autophosphorylation of the β-subunit at tyrosine residues [Y] 1158, 1162, and 1163, the first step in the activation of IR [16]. The level of IR (β subunit) in esophageal tissue of MKR mice was significantly higher (about 2.7-fold) compared with WT mice. We found no significant differences between males and females belonging to the same group (Figure S1A, Supplementary Materials).

The expression level of IGF1R (total protein) in the same tissues was very high (about 10-fold) in MKR esophageal tissue compared to WT expression. We found the highest expression in MKR males although the differences with MKR females did not reach the statistical significance (*p* = 0.141).

Receptor phosphorylation level on tyrosine autophosphorylation sites was evaluated: both IR [pYpY1162/1163] and IGF1R [pYpY1135/1136] expression levels ratio were lower in MKR compared to WT mice of the same gender (Figure S1B, Supplementary Materials). No differences between females and males belonging to the same group were found.

Both, expression and phosphorylation level of some proteins involved in insulin-mediated intracellular signal transduction were analyzed. The activation of PI3K generates phosphatidylinositol (3,4,5)-triphosphate (PIP3), a second messenger that activates 3-phosphoinositide-dependent protein kinase 1 (PDK1) and PDK2, which mediate the effect of insulin on metabolism and cell survival. PDK1 and PDK2, in turn, activate protein kinase B (Akt), by inducing phosphorylation at threonine 308 and serine 473 residues, respectively [16]. Our data showed a significant increase in Akt total protein expression across the four groups (z = +2.06, *p* = 0.040) (Figure S1A). Similarly, Cuzick’s trend test showed a progressive increase in Akt phosphorylation on serine 473 residue across the four groups (z = +2.25, *p* = 0.024), with the highest expression in MKR males (Figure S1B).

After the beta-subunit autophosphorylation step, activated insulin receptor phosphorylates target proteins such as Shc and the family of insulin receptor substrate (IRS) proteins on selective tyrosine residues that serve as docking sites for downstream effector molecules. A lot of studies have focused on Ser/Thr phosphorylation of the IRS proteins as a key negative-feedback control mechanism that uncouples the IRS proteins from their upstream and downstream effectors, and terminates signal transduction in response to insulin, under physiological conditions. Analogous mechanisms are involved on the molecular pathophysiology of insulin resistance by activating serine/threonine IRS kinases that phosphorylate the IRS proteins and inhibit their function. S6K1, a insulin-induced serine/threonine kinase, negatively modulates insulin’s effects by phosphorylating IRS proteins on serine residues [16,17]. We therefore analyzed IRS1 total and inhibited phosphorylated form on serine 307 residue expression in mice esophageal tissue. Figure S1A, showed that in MKR mice IRS1 total expression was up-regulated compared to WT mice. The differences between females groups (MKR females vs. WT females, *p* = 0.004) was statistically significant while the difference between males was close to statistical significance (*p* = 0.031). No evident differences in IRS1 levels were found between males and females belonging to the same group. Data on the relative phosphorylation levels of IRS1 showed a progressive increase across the four groups (z = +3.50, *p* = 5 × 10^−4^) with significant differences also between MKR males and females (*p* = 0.004) (Figure S1B).

Total p70S6K and phosphorylated forms (threonine 421/serine 424) were also evaluated and no difference was detected between the groups (Figure S1A,B).

Together with PI3K/Akt metabolic pathway, the second essential branch of the insulin/IGF-1-signaling pathway is the mitogen-activated protein (MAP) kinase pathway which mediates growth-promoting functions of insulin [16]. To study the state of the mitogenic signaling, total ERK1/2 (Extracellular signal-Regulated Kinase) and phosphorylated ERK1/2 (threonine 185/tyrosine 187) expression were evaluated in the same esophageal tissue. As shown in Figure S1A a significant increase of total ERK1/2 expression across the four groups was found (z = +2.63, *p* = 0.009). The relative phosphorylated form was not significantly altered among the four groups (K-W, *p* = 0.165).

### 2.6. Serum Metabolic Parameters in Operated Mice

To evaluate possible changes that might be related to the onset of the disease, we measured the metabolic parameters in the serum of animals subjected to anastomosis that developed dysplasia or cancer and compared the values with the control groups. Our control group was represented by age (43 weeks)- and sex-matched non-operated animals. We first analyzed the WT animals.

Analysis of serum from wild-type females showed no significant differences in metabolic parameters in the multiple comparisons between non-operated animals and operated animals with dysplastic and/or neoplastic lesions (Table 3). Cuzick’s test trend result showed that glucose levels were increased along with disease progression (z = +2.35, *p* = 0.019).

Sera analysis in wild-type male mice showed a significant difference in leptin level in animals subjected to surgery that developed dysplasia and cancer compared with non-operated controls (Table 3). Cuzick’s test result confirmed that leptin levels decreased with disease progression (z = −2.94, *p* = 0.003). As expected, IL-6 serum level increased along with disease progression (z = +2.01, *p* = 0.044). No significant differences between 13 week- and 43 week-old wild-type animals were found. Mann-Whitney *U*-test showed significantly lower serum insulin concentrations in non-operated WT females compared to WT males at 43 weeks of age (*p* = 0.008) with no significant differences in glucose level (*p* = 0.674).

As showed in Table 4, the Kruskal-Wallis test suggested no significant differences in serum metabolic parameters between non-operated and operated MKR females. All metabolic serum parameters were slightly higher in non-operated MKR females at 43 weeks of age compared to non-operated animals at 13 weeks of age, and the difference in glucose level was statistically significant (Mann-Whitney *U*-test, *p* = 0.016)).

More significant differences came from serum analysis in male MKR group. None of the operated male MKR mice developed dysplasia. Mann Whitney *U*-test performed between 43 weeks old non-operated and operated mice that developed neoplasia showed decreased glucose, C-peptide and leptin levels (Table 4). As expected, in mice that developed cancer IL-6 serum levels were higher compared to non-operated group (Table 4). Non-operated MKR males at 43 weeks of age had higher levels C-peptide compared to non-operated MKR males at 13 weeks of age (Mann-Whitney *U*-test, *p* = 0.010). Furthermore, Mann-Whitney *U*-test showed significant differences in insulin levels between non-operated MKR females and MKR males at 43 weeks of age (*p* = 0.008).

In the end, we analyzed the trend of each serum parameter across the four groups and on the basis of histology. Across non-operated groups we found significant insulin (Cuzick z = +5.16, *p* < 1·10^−4^) and C-peptide increased levels (Cuzick z = +5.04, *p* < 1 × 10^−4^) and leptin decreased level (Cuzick z = −3.34, *p* = 8 × 10^−4^). No significant differences in glucose and IL-6 levels were found. The analysis of the trend across mice that developed dysplasia was performed across three group (MKR males did not develop dysplasia) and showed, in parallel with the healthy mice, increased insulin (Cuzick z = +3.51, *p* = 4 × 10^−4^) and C-peptide serum levels (Cuzick z = +3.28, *p* = 0.001). No differences on the other parameters were found. Serum analysis of mice that developed cancer showed, in parallel with the others, increased insulin (z = +5.06, *p* < 1 × 10^−4^) and C-peptide levels (z = +4.42, *p* < 1 × 10^−4^) and decreased glucose and leptin levels (z = −2.89, *p* = 0.004 and z = −2.99, *p* = 0.003, respectively). IL-6 levels were comparable (z = +0.88, *p* = 0.380).

### 2.7. Insulin Signaling in Operated Mice Esophageal Tissue

**Figure 2:**
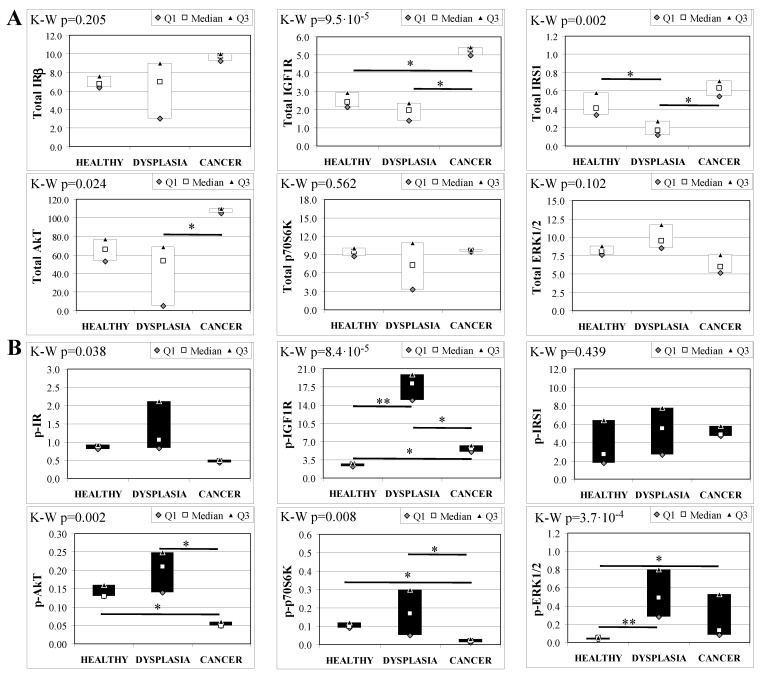
Insulin signaling in operated WT female mice esophageal tissue. Data are represented as Median (Q1; Q3). Data obtained in operated mice affected by dysplasia or cancer were compared to strain-, sex-, and age-matched, non-operated animals (43 weeks). K-W indicates the Kruskal-Wallis test result, used for multiple comparisons among the three groups of mice. The Mann-Whitney U-test was performed to compare data distribution between two groups of mice. After correction a A *p*-value lower than 0.016 (Bonferroni adjustment) was considered statistically significant: * *p* < 0.016; ** *p* < 1.6× 10^−3^. (**A**) Total protein expression: the quantity of each total protein considered was expressed in ng/100 mg of total protein extracted by the distal mice esophageal segment. (**B**) Phosphorylated protein expression: the amount of the six phospho-proteins considered was expressed as phosphorylated Units for each ng of the corresponding total protein amount. Proteins were quantified by Luminex-xMAP Technology on the basis of a standard curve. Beta-tubulin expression was used as the internal loading control.

**Figure 3:**
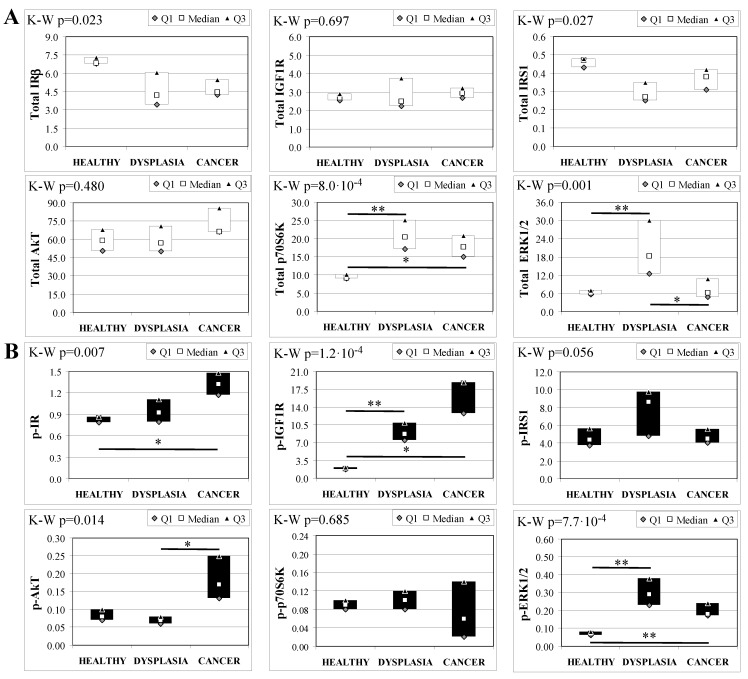
Insulin signaling in operated WT male mice esophageal tissue. Data are represented as Median (Q1; Q3). Data obtained in operated mice affected by dysplasia or cancer were compared to strain-, sex-, and age-matched, non-operated animals (43 weeks). K-W indicates the Kruskal-Wallis test result, used for multiple comparisons among the three groups of mice. The Mann-Whitney U-test was performed to compare data distribution between two groups of mice. A *p*-value lower than 0.016 (Bonferroni adjustment) was considered statistically significant: * *p* < 0.016; ** *p* < 1.6 × 10^−3^. (**A**) Total protein expression: the quantity of each total protein considered was expressed in ng/100 mg of total protein extracted by the distal mice esophageal segment. (**B**) Phosphorylated protein expression: the amount of the six phospho-proteins considered was expressed as phosphorylated Units for each ng of the corresponding total protein amount. Proteins were quantified by Luminex-xMAP Technology on the basis of a standard curve. Beta-tubulin expression was used as the internal loading control.

To evaluate the involvement of insulin and IGF receptors in the oncogenic process during esophageal cancer onset related to duodeno-esophageal chronic reflux condition, insulin signaling on the esophageal tissue of operated animals was analyzed. Also in this case, the control groups included strain-, age-, and sex-matched, non-operated animals. Analysis of the esophageal tissue of WT females (Figure 2) showed a significantly higher phosphorylation level of IGF1R in both dysplastic (7-fold higher) and cancer (2.2-fold higher) tissues compared to healthy tissue. IGF1R hyperactivation could lead to the increase in ERK1/2 protein phosphorylation (10-fold and 2.6-fold in dysplastic and cancer tissue, respectively), compared with basal state (Figure 2B). Total IGF1R expression was up-regulated in cancer tissue compared to healthy tissue. Moreover, activation of p70S6K was found in dysplastic tissue, as well as an increase in IRS1 total protein degradation. Tissue from WT males (Figure 3) showed a higher phosphorylation state of both IR and IGF1R in cancer tissue compared with healthy non-operated esophageal tissue (Figure 3B). Cuzick’s trend test showed that phosphorylation increased in parallel with disease progression (z = +3.13, *p* = 0.002 for IR phosphorylation and z = +4.16, *p* < 1 × 10^−5^ for IGF1R phosphorylation, respectively). In parallel, increased ERK1/2 phosphorylation levels were found in both dysplastic and cancer tissues compared with control tissues. Total IR expression level in cancer tissues from WT males was decreased compared with healthy tissue (Figure 3A). The data were confirmed by the Cuzick’s trend test result, which showed a significant decrease in IR expression along with disease progression (z = −2.26, *p* = 0.024). As expected, increased Akt phosphorylation levels were found in cancer tissue. Unlike in WT females, higher levels of p70S6K were found in both dysplastic and neoplastic lesions in WT males. Cuzick’s trend test showed an increase of p70S6K expression along with disease progression (z = +2.85, *p* = 0.004).

**Figure 4:**
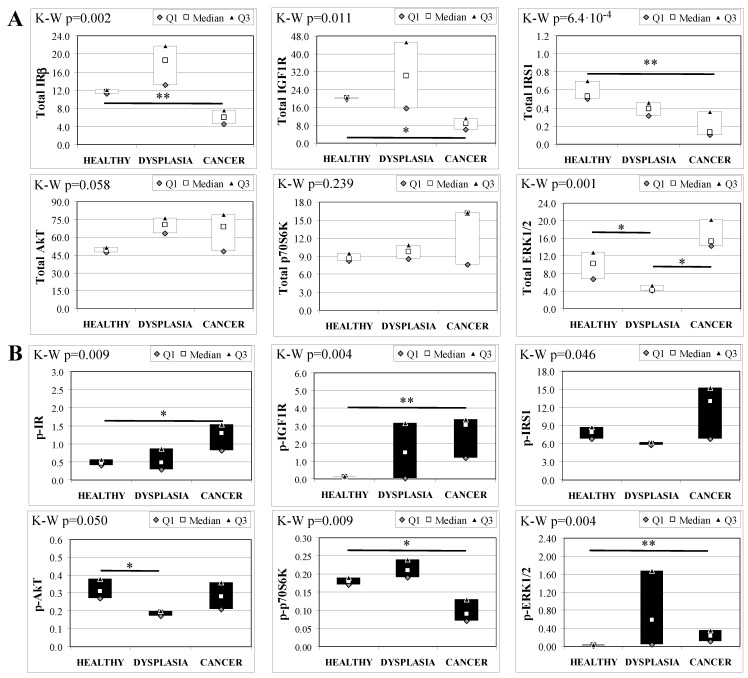
Insulin signaling in operated MKR female mice esophageal tissue. Data are represented as Median (Q1; Q3). Data obtained in operated mice affected by dysplasia or cancer were compared to strain-, sex-, and age-matched, non-operated animals (43 weeks). K-W indicates the Kruskal-Wallis test result, used for multiple comparisons among the three groups of mice. Mann-Whitney U-test was performed to compare data distribution between two groups of mice. A *p*-value lower than 0.016 (Bonferroni adjustment) was considered statistically significant: * *p* < 0.016; ** *p* < 1.6 × 10^−3^. (**A**) Total protein expression: the quantity of each total protein considered was expressed in ng/100 mg of total protein extracted by the distal mice esophageal segment. (**B**) Phosphorylated protein expression: the amount of the six phospho-proteins considered was expressed as phosphorylated Units for each ng of the corresponding total protein amount. Proteins were quantified by Luminex-xMAP Technology on the basis of a standard curve. Beta-tubulin expression was used as the internal loading control.

Esophageal tissue analysis from MKR females showed statistically significant differences in most of the proteins analyzed (Figure 4). IR, IGF1R and IRS1 total protein expression decreased along with disease progression, as confirmed by Cuzick’s test for trend (z = −3.07, *p* = 0.002; z = −2.74, *p* = 0.006 and z = −3.71, *p* < 1 × 10^−5^, for IR, IGF1R and IRS1, respectively) (Figure 4A). On the other hand, the relative phosphorylation levels of both IR and IGF1R significantly increased along with disease progression (z = +2.92, *p* = 0.004 for phospho-IR; z = +3.21, *p* = 0.001 for phospho-IGF1R) (Figure 4B). Akt total protein expression was higher in dysplastic and neoplastic lesions although the differences did not reach statistical significance. Similarly, p70S6K total protein expression increased in both dysplastic and neoplastic tissues but the trend did not reach statistical significance (z = +1.64, *p* = 0.108). On the contrary, the phosphorylation state of p70S6K was decreased along with disease progression (z = −2.32, *p* = 0.020). ERK1/2 phosphorylation level was higher in cancer tissues compared with healthy esophagus. Cuzick’s trend test showed that phospho-ERK1/2 expression increased along with disease progression (z = +3.10, *p* = 0.002). ERK1/2 total expression was higher in cancer tissue compared to control tissue and the differences were close to statistical relevance (M–W, *p* = 0.021).

**Figure 5:**
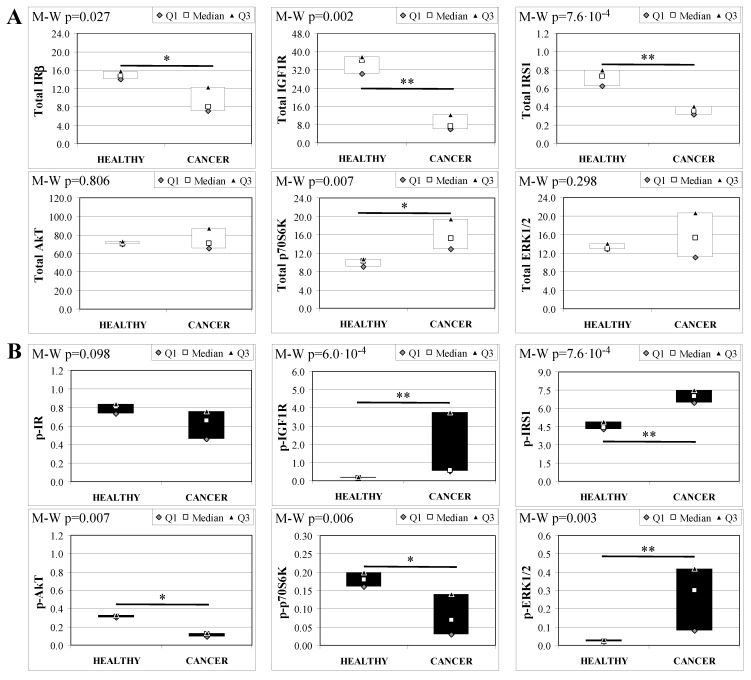
Insulin signaling in operated MKR male mice esophageal tissue. Data are represented as Median (Q1; Q3). Data obtained in operated mice affected by cancer were compared to strain-, sex-, and age-matched, non-operated animals (43 weeks). The Mann-Whitney U-test was performed to compare data distribution between the two groups of mice. A *p*-value lower than 0.05 was considered statistically significant: * *p* < 0.05; ** *p* < 0.005. (**A**) Total protein expression: the quantity of each total protein considered was expressed in ng/100 mg of total protein extracted by the distal mice esophageal segment. (**B**) Phosphorylated protein expression: the amount of the six phospho-proteins considered was expressed as phosphorylated Units for each ng of the corresponding total protein amount. Proteins were quantified by Luminex-xMAP Technology on the basis of a standard curve. Beta-tubulin expression was used as the internal loading control.

Analogous to MKR females, protein expression in esophageal tissue from MKR males showed a significant decrease of both IR and IGF1R expression in cancer compared with control tissue (Figure 5A). Also in this group, IGF1R phosphorylation was increased in cancer tissue (Figure 5B). The p70S6K expression was increased, as well as IRS1 phosphorylation and degradation. Surprisingly, the phosphorylation of AkT protein was significantly reduced in cancer tissue. Mitogenic pathway activation was found to be higher in cancer, with a phosphorylation level of ERK1/2 15-fold higher in cancer than control tissue.

At the end, we analyzed the trend of each protein amount across the four groups and on the basis of histology. Across non-operated groups we found a significant up-regulation of IR (Cuzick z = +4.92, *p* < 1·10^−4^), IGF1R (Cuzick z = +5.01, *p* < 1 × 10^−4^), and ERK1/2 (Cuzick z = +3.08, *p* = 0.002) total protein expression. Phospho-p70S6K expression was increased across the four groups (Cuzick z = +3.44, *p* = 6 × 10^−4^), and in parallel, relative phospho-IGF1R expression was decreased (Cuzick z = −4.19, *p* < 1 × 10^−4^).

Comparing protein expression on dysplastic tissues, the increase of IGF1R total protein expression and the decrease of relative phospho-IGF1R level were confirmed by the trend test (Cuzick z = +3.45, *p* = 6 × 10^−4^ for IGF1R; z = −3.71, *p* = 2 × 10^−4^ for phospho-protein). When we evaluated protein expression on cancer tissues, the data confirmed the same trend (Cuzick z = +3.05, *p* = 0.002 for IGF1R and z = −3.60, *p* = 3 × 10^−4^ for phospho-protein) and also showed an increased expression of total ERK1/2 (Cuzick z = +3.04, *p* = 0.002) across the groups.

### 2.8. Ki-67 Nuclear Protein Expression on Esophageal Lesions

To evaluate whether IGF1R increased expression was related to cellular proliferation pathway induction, Ki-67 nuclear protein expression was analyzed on esophageal tissue specimens.

**Figure 6:**
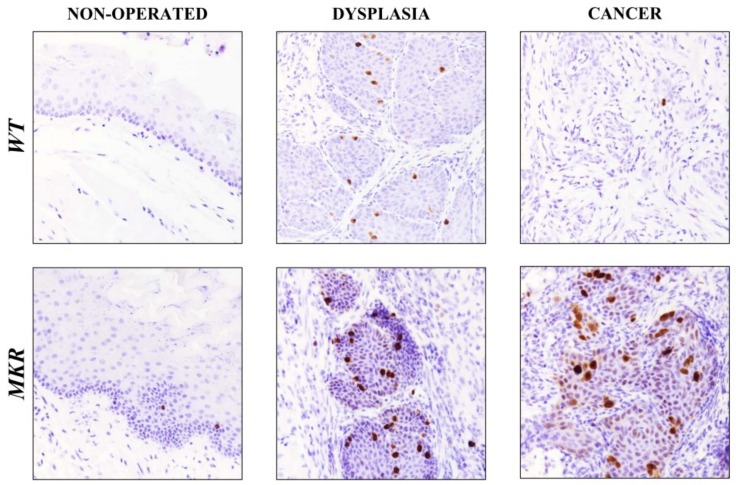
Representative case of Ki-67 nuclear protein expression on healthy, dysplastic and neoplastic esophageal tissues in WT and MKR mice. Non-operated mice (healthy tissue): WT male and MKR male at 43 weeks of age. Dysplasia: squamous high-grade dysplastic lesions (HGD) in WT female and MKR female. Cancer: Moderately well differentiated ESCC in WT male and MKR male. Original magnification 40×.

As showed in Figure 6, in dysplastic lesions the average number of Ki-67 positive cells was 17.5 (±3.5) and 25.5 (±2.7) in WT and MKR tissues, respectively, while 1.5 (±0.8) and 26.4 (±5.4) positive cells were found in WT and MKR neoplastic lesions, respectively.

### 2.9. HER-2 Expression in Cancer Progression

On the basis of our previous retrospective study [18], we hypothesized the involvement of other growth factor receptors and their cooperation with the insulin/insulin-like growth factor-1 axis in the evolution of these tumors. HER2 overexpression plays a critical role in the development, progression and metastasis of many malignancies such as breast cancer [12,13,19], gastric cancer [20,21] and esophageal cancer [18,22]. To evaluate whether in our carcinogenesis model (related exclusively to duodenal content chronic reflux) that HER2 could play a role, HER2 total protein expression was evaluated in esophageal tissue on both non-operated and operated mice.

**Figure 7:**
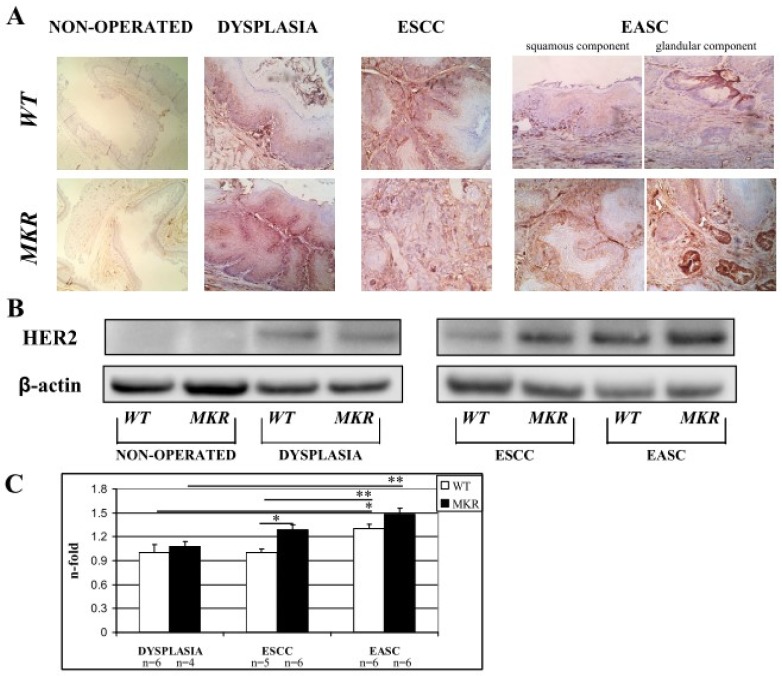
Representative case of HER2 overexpression detected by Immunohistochemistry IHC (**A**) and Western blotting (**B**). (**A**) WT (wild-type mouse esophageal tissue). From left to right: 1. Non-operated (healthy tissue) WT female at 43 weeks of age (IHC negative; original magnification 100×). 2. Squamous Dysplasia (LGD/HGD) in WT male (IHC2+; original magnification 200×). 3. ESCC in WT male (IHC3+ on both dysplastic and infiltrative component; original magnification 200×). 4. EASC in WT male (IHC2+ in squamous component and IHC1+ in glandular component; original magnification 200×). MKR (MKR mouse esophageal tissue). From left to right: 1. Non-operated (healthy tissue) MKR female at 43 weeks of age (IHC negative; original magnification 100×). 2. Squamous Dysplasia (HGD) in MKR female (IHC3+; original magnification 200×). 3. ESCC in MKR female (IHC2+; original magnification 200×). 4. EASC in MKR male (IHC3+ in both squamous and glandular component; original magnification 200×). (**B**) Thirty micrograms of extracted total proteins were separated in SDS-PAGE and transferred to nitrocellulose membranes. Membranes were blotted with anti-HER2 (molecular weight = 185 KDa) and anti-β-actin (molecular weight = 42 KDa) antibodies. Non-operated (healthy tissue) WT and MKR male at 43 weeks of age. Dysplasia: squamous HGD dysplasia in WT and MKR female. ESCC in WT and MKR female. EASC in WT and MKR male. (**C**) The densitometric intensity of bands is shown in the bar graphs. Data are presented as the mean ± SEM and the relative protein expression is indicated as *n*-fold of protein amount detected on WT dysplastic tissue which was set equal to 1 arbitrary unit. * *p* < 0.05; ** *p* < 0.01.

Immunohistochemistry staining of FFPE esophageal tissues showed HER2 positivity in both dysplastic and neoplastic lesions in ESCC and EASC, in WT and MKR mice, as previously described in human specimens [18,22,23]. As shown in the representative images (Figure 7A) all dysplastic and neoplastic lesions showed HER2 positivity. Data were confirmed also by Western blotting analysis (Figure 7B). Densitometric analysis (Figure 7C) showed a higher HER2 expression in neoplastic lesions compared to dysplastic lesions of both WT and MKR mice. Furthermore, in ESCC tissue from MKR mice, HER2 expression was significantly higher (30% more) that in ESCC WT tissue.

## 3. Discussion

Esophageal cancer is the sixth most common cause of cancer-related mortality in the world [24]. This malignancy exists in two main forms with distinct etiological and pathological characteristics, esophageal squamous cell carcinoma (ESCC) and esophageal adenocarcinoma (EAC) [25].

It is widely accepted that ESCC is associated with smoking and alcohol consumption [26]. EAC is the most common histological subtype of esophageal cancer in the west [27]. Barrett’s esophagus (BE) is the only known precursor for EAC and develops in patients with gastroesophageal reflux disease [28,29]. It is reported that not only gastric acid, but also the reflux of duodenal content plays an important role in the generation of Barrett’s metaplasia and EAC [30].

Previous studies have demonstrated that refluxed duodenal contents cause esophageal carcinoma in rats without exposure to carcinogens [31,32,33]. The histological spectrum of these carcinomas includes ESCC, EAC and adenosquamous carcinoma [25].

With the total exclusion of the stomach, our mouse surgical model showed that refluxed duodenal content caused esophageal cancer without exposure to carcinogens, and without any prevalence between the two tumor histological types (47.4% were ESCC and 52.6% were EASC). It is unclear what factors lead to the formation of carcinomas of specified histology, but in our model, adenosquamous carcinoma seems to be related to male gender.

The first important result that came from this work is that the hyperinsulinemic condition in MKR mice increases more than six-fold the risk of developing neoplastic esophageal lesions in the presence of duodenal chronic reflux.

The metabolic status analysis of the mice before surgery showed, as expected, that MKR mice have higher serum insulin and C-peptide levels compared to age- and sex-matched WT mice. Body weight was sensibly lower in MKR mice compared to the WT counterpart which tended to accumulate more adipose tissue than MKR mice, as confirmed by leptin serum level measurements. Both young and older WT males accumulated more adipose tissue and had higher insulin levels than females WT counterparts. Differences in insulin levels between MKR males and females were especially evident in older age (43 weeks). The highest insulin secretion in MKR male mice suggested that males were affected by a more severe insulin-resistance that could evolve towards an overt diabetes. These gender-specific differences may be attributed to estrogens, which exert a protective effect on pancreatic β-cells [34]. Moreover, it is known that male sex is a risk factor for the onset of this type of cancer and our results confirmed this epidemiological data. On the basis of insulin and C-peptide serum levels, the four groups of animals were ordered lowest to highest as follows: WT females, WT males, MKR females and MKR males.

Insulin and insulin-like growth factor 1 are closely related hormones that control different aspects of growth and metabolism in many organisms [35,36,37]. At physiological levels, insulin and IGF-1 fully activate their cognate receptors, IGF-1 can also activate IR/IGF1R hybrid receptors, and at supraphysiological levels they can also bind and activate the other receptor in cell culture models, although with reduced affinity [38]. Today it is known that IGF1R is involved in mitogenesis, transformation and protection from apoptosis [39,40]. A pivotal role of IGF1R signaling in esophageal cancer onset in our animal model was first suggested by the high activation level of the IGF1R in dysplastic and cancer tissues of WT normoinsulinemic mice, suggesting that IGF1R is involved in duodenal reflux-dependent esophageal carcinogenesis even in the absence of hyperinsulinemia.

The hyperinsulinemic condition of MKR mice is responsible for an important alteration of the expression and activation of IR/IGF1R also in esophageal tissue which is not a classical target tissue of insulin action, as demonstrated by the data from MKR non-operated animals. Our data showed that hyperinsulinemia was responsible for an increase in both metabolic and mitogenic insulin signal transduction as confirmed by the significant increase in Akt phosphorylation level, higher inhibitory IRS1 phosphorylation (as an attempt to switch-off an over-activated insulin signal) and an increase in ERK1/2 expression. These molecular alterations were increased across the four ordered groups. This evidence might explain the differences in tumor incidence observed between females (WT = 30.0%, MKR = 55.5%,) and males (WT = 33.3%, MKR = 94.4%) in both groups. The IGF1R over-expression was observed in all cases, healthy, dysplastic and cancer tissue compared to WT tissue. The over-expression in dysplasia and cancer was related to cellular proliferation pathway induction increase in MKR lesions. Importantly, cancer tissue analysis showed a very small amount of insulin and IGF1 receptors compared to non-operated or dyplastic tissue or both in all groups of animals, with the exception of WT females. Our study is not unique in finding lower growth factor receptor expression levels in tumor tissue; in breast and colorectal cancer; this phenomenon has also been described and it seems to be related to the advanced and poorly differentiated carcinoma [41,42]. We showed similar results in esophageal adenocarcinoma tissue from patients analyzed in our retrospective study published last year [18]. In the present study, we showed an important activation of p-ERK1/2 in both dysplastic and cancer MKR esophageal tissue, which is known to be a mitogenic signaling protein down-stream of IGF1R activation. In an attempt to clarify IGF1R regulation in duodenal reflux-induced esophageal carcinogenesis we hypothesized a receptor cross-talk between IGF1R and another crucial growth factor receptor that is known to have a pivotal role in EAC carcinogenesis and disease progression, such as Human Epithermal Growth Factor receptor 2, HER2. Our group together with other studies showed HER2 over-expression on human Barrett’s tissue and esophageal adenocarcinoma [18,22,43]. In our previous study [18] in vitro experiments on OE19 human esophageal adenocarcinoma cell line were performed. HER2 expression and phosphorylation levels resulted to be significantly up-regulated after insulin treatment. In the same set of experiments, insulin-induced cell proliferation was inhibited by Trastuzumab, a humanized monoclonal antibody against HER2 receptor successfully used in early-stage and metastatic breast cancer therapy of patients with HER2-overexpressing tumors, suggesting a cross-talk between insulin and HER2 signals. Here, our hypothesis could be supported by immunohistochemical and biochemical analyses confirming that both, dysplastic and cancer tissues over-expressed HER2. Moreover, the hyperinsulinemic condition increased HER2 cancer expression. A cross talk between the ErbB/HER family and IGF1R signaling pathway has been proven in breast cancer [11,12,13] and the evidences reported in this study supports the hypothesis that this mechanism could be also involved in this context.

## 4. Materials and Methods

### 4.1. Animals

Procedures involving animals and their care were conducted according to Italian law on the use of experimental animals (DL n. 116/92 art. 5). This study was approved by the Ethical Committee of University of Padova (Comitato Etico di Ateneo sulla Sperimentazione Animale, Protocol n. 32140, Project n. 40/2011, approved on June 14, 2011). All animals were housed and maintained in an animal facility at the Veneto Institute of Oncology (Istituto Oncologico Veneto-IOV-IRCCS). Mice were kept on a 12-h light/dark cycle with access to a standard laboratory chow diet and fresh water ad libitum. All the mice used in this study were on the FVB/N background, wild-type (WT) and transgenic, hyperinsulinemic, insulin-resistant MKR^+/+^ (MKR) mice.

### 4.2. Surgical Procedure

At 13 weeks of age, 39 WT (*n* = 20 females, *n* = 19 males) and 40 MKR (*n* = 20 females, *n* = 20 males) mice underwent jejunum-esophageal anastomosis. The operation to induce chronic duodeno-esophageal reflux was performed according to the microsurgical procedure described in our previous studies [43,44] with some modification. Briefly, following induction of anesthesia with intraperitoneal injection (tiletamine/zolazepam (25 mg/Kg) and xylazine (2 mg/Kg)), a 2-cm midline laparotomy incision was made. The stomach was completely closed with a ligature; a loop of jejunum, 2 cm distal to the ligament of Treitz, was anastomozed side-to end to the esophago-gastric junction, leaving the stomach in situ. Postoperatively, the animals had free access to water and food. Thirteen-week-old and 43-week-old non-operated WT (*n* = 8, for each gender and for each age) and MKR (*n* = 8, for each gender and for each age) were used as control mice.

### 4.3. Tissues Collection

Thirty weeks after surgery, blood samples were recovered through intracardiac aspiration on 2-hr fasted mice. After euthanasia, the entire esophagus was removed and opened longitudinally to examine macroscopic morphological changes. The tissue was divided longitudinally: one portion was fixed in 4% buffered formalin and embedded in paraffin for histological and immunohistological assessment, and the remaining longitudinal strip was divided into the three esophageal segments (proximal, medial and distal), frozen in liquid nitrogen and it was stored at −80 °C for subsequent protein expression analysis.

### 4.4. Histological and Immunohistochemical Analysis

FFPE (Formalin-Fixed, Paraffin-Embedded) samples were cut serially (4 μm thick) and stained with haematoxylin and eosin. Two experienced gastrointestinal pathologists (Cinzia Giacometti, Matteo Fassan) reviewed all the slides in a blinded fashion. On the FFPE samples, immunohistochemical analysis for HER2 was performed with the Oracle HER2 Bond IHC system (CB11) mouse monoclonal antibody (Menarini Diagnostics, Florence, Italy), whilst immunohistochemical analysis for Ki-67 was performed with anti-Ki-67 monoclonal antibody (clone SP6) (Spring Bioscience Corporation, Pleasanton, CA, USA) using the automated Leica Microsystems Bond-Max (Leica, Wetzlar, Germany). The number of immunoreactive Ki-67 cells was determined as the average of the positive immunostained cells in five fields (magnification 40×) selected randomly on three slides for each tissue sample.

### 4.5. Serum Analysis

Blood glucose (mg/dL) was determined on fresh blood samples with a glucometer (Abbott Laboratories^®^, Irving, TX, USA). Serum was extracted and frozen in liquid nitrogen. Insulin (pg/mL), C-peptide (pg/mL), leptin (pg/mL) and IL-6 (pg/mL) levels were simultaneously measured in each serum sample with Luminex^®^ xMAP^®^ Technology (Merck KGaA, Darmstadt, Germany). Each measure was performed in duplicate. Serum analyte quantitative analyses were performed with Luminex x PONENT 3.1 Software (Luminex Corporation, Austin, TX, USA) using a Five Parameter Logistic curve fitting.

### 4.6. Esophageal Tissue Analysis

Total proteins were obtained from fresh frozen distal esophageal segments. After mechanical lysis tissue fragments were treated with NP40 Lysis Buffer added with protease and phosphatase inhibitors (Thermo Scientific Pierce, Rockford, IL, USA). Bicinchoninic acid (BCA) assay (Thermo Scientific Pierce, Rockford, IL, USA) was performed to quantify extracted total proteins. For each sample, 20 μg of total proteins were analyzed. Signaling pathways for IR/ IGF1R were determined using Luminex xMAP^®^ technology. Each measure was performed in duplicate.

### 4.7. Immunoblotting

Thirty µg of extracted total proteins were separated in SDS-PAGE and transferred to nitrocellulose membranes. After being blocked with TBS-T (150 mM NaCl, 50 mM Tris-HCl pH 7.4, 0.1% Tween-20) supplemented with 5% BSA (Bovine Serum Albumin), membranes were blotted with anti-HER2 monoclonal antibody (clone D8F12) (Cell Signaling Technology, Danvers, MA, USA). Moreover, membranes were blotted with an anti-β-actin polyclonal antibody (Sigma–Aldrich, St. Louis, MO, USA) used as a control for equal loading. Images were acquired and digitally scored with a densitometer image analyzer (Quantity one software, Bio-Rad, Hercules, CA, USA). Four independent samples were analyzed for each group of non-operated animals. Densitometric analysis was performed normalizing protein expression to β-actin expression. Data were presented as the mean ± SEM (Standard Error of the Mean). Data on dysplastic tissue represented the mean of *n* = 6 and *n* = 4 independent samples from WT and MKR mice, respectively. Data on ESCC tissue represented the mean of *n* = 5 and *n* = 6 independent samples from WT and MKR mice, respectively. Data on EASC tissue represented the mean of six independent samples for each group of animals. Relative protein expression was represented as n-fold of protein amount detected on WT dysplastic tissue which was set equal to 1 arbitrary unit.

### 4.8. Statistical Analysis

Fisher’s exact test was used for comparison of categorical variables. The Kruskal-Wallis test was used for multiple comparisons. A *p*-value lower than 0.05 was considered statistically significant. The Mann-Whitney U-test, followed by Bonferroni correction, was used to assess differences between two groups of mice. In the case of multiple comparisons among four groups, following Bonferroni correction, the p value was adjusted at 0.008. In the case of multiple comparison among three groups, the Bonferroni-adjusted p value was 0.016. On the basis of insulin secretion, mice groups were expressed as an ordered categorical variable. Cuzick’s test for trend was used to evaluate the trend of protein expression across the four ordered groups: WT females, WT males, MKR females, MKR males. Cuzick’s test for trend was also used to evaluate protein expression along with disease severity progression: non-operated (healthy tissue), dysplastic, and neoplastic lesions.

The amount of the specific total proteins from esophageal tissue was calculated on the basis of a standard curve with known concentration. Beta-tubulin amount was used as internal loading control.

Protein was expressed as ng per 100 μg of total protein loaded. Phosphorylated protein expression was evaluated as a ratio between the phosphorylated protein amount/ng of total corresponding protein. Beta-tubulin amount was used as an internal loading control.

Data were expressed as a median and interquartile range (Q1; Q3). Only the data referred to in the densitometric analysis of immunoblotting were expressed as mean ± SEM, and in this case the Student’s *t* test was used to assess differences between groups. A *p*-value lower than 0.05 was assumed to indicate a significant difference. Data analyses were performed with SPSS v20 and Stats-Direct.

## 5. Conclusions

**Figure 8:**
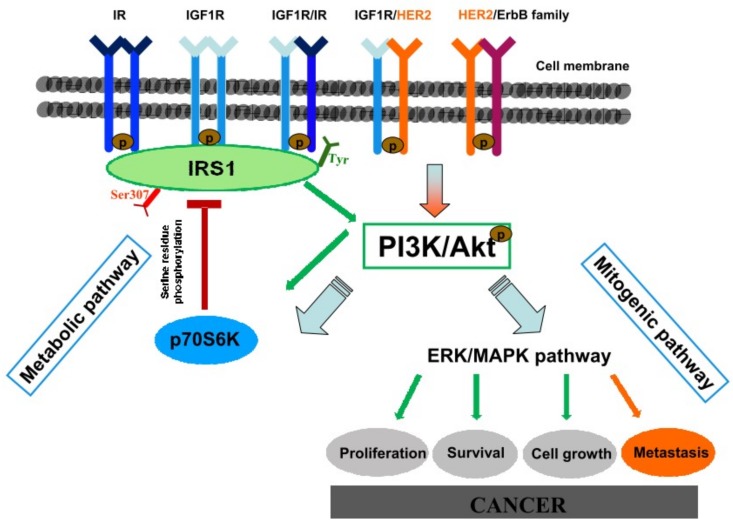
Hypothesized hyperinsulinemia-induced cancerogenetic mechanisms. Insulin and insulin-like growth factor 1 are closely related hormones that control different aspects of growth and metabolism in many organisms. At physiological levels, insulin and IGF-1 fully activate their cognate receptors and IGF-1 can also activate IR/IGF1R hybrid receptors. At supraphysiological levels they can also bind and activate each other’s’ receptor in cell culture models, although with reduced affinity. Activated IR/IGF1R receptors phosphorylate target proteins such as Insulin Receptor Substrate (IRSs) proteins on selective tyrosine residues (Tyr) that serve as docking sites for downstream effector molecules. Phosphorylated IRS1 activates the metabolic PI3K/Akt pathway. Activated Akt regulates glucose metabolism and induces the mitogenic (ERK/MAPK) pathway involved in carcinogenic mechanism. Serine (Ser)/Threonine phosphorylation of the IRS proteins is a key negative-feedback control mechanism that uncouples the IRS proteins from their upstream and downstream effectors and terminates signal transduction in response to insulin, under physiological conditions. Analogous mechanisms are involved in the molecular pathophysiology of insulin resistance by activating serine/threonine IRS kinases (such as the p70S6K) that phosphorylate the IRS proteins and inhibit their function. Hyperinsulinemia is responsible of receptors hyperactivation inducing IR, IGF1R, and ERK over-expression. HER2 signal activation is known to have a pivotal role in esophageal cancerogenesis and disease progression. Hyperinsulinemia is able to induce also HER2 overexpression. HER2 is the preferential dimerization partner of other members of the ErbB family and other growth factor receptors. A cross-talk between HER2 and IGF1R signaling pathway has been proven in breast cancer through an heterodimerization mechanism. An analogous mechanism is proposed also in this context, in which hyperinsulinemia-induced IGF1R overexpression might enhance the possibility to form HER2/IGF1R heterodimers, promoting mitogenic pathway activation and, in turn, cancer.

In conclusion, the reflux of duodenal contents has a great potential for malignant initiation, inducing not only EASC but also ESCC. IGF1R activation seems to play a crucial role in esophageal carcinogenesis in this animal model. Hyperinsulinemia significantly increases reflux-related esophageal cancer incidence, possibly through enhancing IGF1R/p-ERK1/2 pro-proliferation signaling in esophageal cells. Because of the pivotal role of HER2 in esophageal carcinogenesis and the possibility of heterodimers formation between IGF1R and HER2, we suggest that a cross talk between the ErbB/HER family and IGF1R signaling pathway could sustain IGF1R activation and down-stream p-ERK1/2 mediated proliferation, in particular in hyperinsulinemic MKR mice in which IGF1R is highly expressed even in absence of lesions, and in which the higher insulin concentration induces cancer cell HER2 over-expression (Figure 8).

## Figures and Tables

**Table 1:** Incidence of esophageal pathological changes in wild-type (WT) and hyperinsulinemic (MKR) mice with duodenal reflux. Data show the numbers of mice, with percentages in parentheses.

Group	Lesion			
	No Lesion	Esophagitis	Dysplasia	Cancer
WT (*n* = 38)	2 (5.3)	0 (0.0)	24 (63.1)	12 (31.6)
MKR (*n* = 35)	1 (2.9)	4 (11.4)	4 (11.4)	26 (74.3)

**Table 2:** Incidence of esophageal pathological changes in wild-type (WT) females and males and hyperinsulinemic (MKR) females and males mice with duodenal reflux. Data show the numbers of mice, with percentages in parentheses. Animals were divided by gender; dysplastic lesions were divided based on the degree of histologic abnormality in LGD (Low-Grade Dysplasia) and HGD (High-Grade Dysplasia); neoplastic lesions were divided by histotype in ESCC (Esophageal Squamous Cell Carcinoma) and EASC (Esophageal Adeno-Squamous Carcinoma).

Group	Gender	No Lesion	Esophagitis	LGD	HGD	ESCC	EASC
WT (*n* = 38)	FEMALE (*n* = 20)	1 (5.0)	0 (0.0)	11 (55.0)	2 (10.0)	4 (20.0)	2 (10.0)
	MALE (*n* = 18)	1 (5.5)	0 (0.0)	5 (27.8)	6 (33.4)	1 (5.5)	5 (27.8)
MKR (*n* = 35)	FEMALE (*n* = 18)	0 (0.0)	4 (22.2)	2 (11.1)	2 (11.1)	6 (33.4)	4 (22.2)
	MALE (*n* = 17)	1 (5.9)	0 (0.0)	0 (0.0)	0 (0.0)	7 (41.2)	9 (52.9)

**Table 3:** Serum metabolic parameters in operated WT mice. Data were expressed as Median (Q1; Q3). Data obtained in operated mice affected by dysplasia or cancer were compared to strain-, sex-, and age-matched non-operated animals (43 weeks). K–W test indicates the Kruskal-Wallis test result used for multiple comparisons among the three groups of mice. A *p*-value lower than 0.05 was considered statistically significant.

**WT** **Females**	**Non-Operated** ***n* = 8**	**Dysplasia** ***n* = 13**	**Cancer** ***n* = 6**	**K–W** **Test**
**Glucose** (mg/dL)	177.5 (147.3; 192.3)	196.0 (125.0; 254.0)	235.5 (225.3; 291.5)	0.179
**Insulin** (pg/mL)	423.4 (374.2; 462.2)	376.5 (360.0; 390.0)	443.5 (420.0; 741.8)	0.215
**C-Peptide** (pg/mL)	323.0 (295.2; 423.9)	313.3 (240.1; 405.8)	439.0 (346.9; 522.5)	0.334
**Leptin** (pg/mL)	2345.8 (1998.7; 2912.6)	1928.0 (1515.7; 2540.0)	1784.5 (1640.1; 1962.0)	0.103
**IL-6** (pg/mL)	23.2 (3.1; 31.9)	29.0 (1.4; 55.2)	59.8 (21.8; 74.3)	0.292
**WT** **Males**	**Non-Operated** ***n* = 8**	**Dysplasia** ***n* = 11**	**Cancer** ***n* = 6**	**K–W** **Test**
**Glucose** (mg/dL)	191.0 (166.3; 208.3)	209.0 (175.3; 252.8)	222.0 (157.3; 242.5)	0.469
**Insulin** (pg/mL)	838.7 (689.6; 860.4)	784.0 (467.5; 973.1)	1406.4 (1033.8; 1912.6)	0.094
**C-Peptide** (pg/mL)	761.7 (407.5; 922.5)	661.2 (334.9; 755.4)	850.2 (814.3; 1060.2)	0.210
**Leptin** (pg/mL)	4902.2 (3801.4; 6749.4)	1564.8 (1336.5; 2811.2)	1104.1 (894.0; 1415.5)	0.011
**IL-6** (pg/mL)	24.9 (13.4; 28.3)	29.0 (11.1; 40.7)	65.8 (37.4; 162.7)	0.121

**Table 4:** Serum metabolic parameters in operated MKR mice. Data were expressed as Median (Q1; Q3). Data obtained in operated mice affected by dysplasia or cancer were compared to strain-, sex-, and age-matched non-operated animals (43 weeks). K–W test indicates the Kruskal-Wallis test result used for multiple comparisons among the three groups of mice. M–W test indicates Mann-Whitney *U*-test result used to compare data distribution between two groups of mice (No MKR males were affected by dysplastic lesions). A *p*-value lower than 0.05 was considered statistically significant.

**MKR** **Females**	**Non-Operated** ***n* = 8**	**Dysplasia** ***n* = 4**	**Cancer** ***n* = 10**	**K–W** **Test**
**Glucose** (mg/dL)	188.0 (169.0; 206.8)	258.0 (220.3; 302.0)	219.5 (201.3; 241.3)	0.102
**Insulin** (pg/mL)	2713.9 (2266.0; 5179.1)	4884.8 (4595.4; 5084.0)	3160.0 (2160.8; 3517.8)	0.055
**C-Peptide** (pg/mL)	1498.9 (1182.9;1729.7)	1711.2 (1496.1;1926.5)	1534.4 (1074.7;2560.9)	0.919
**Leptin** (pg/mL)	1548.9 (968.1;1975.2)	1323.0 (910.8;1565.1)	921.1 (842.0;1123.9)	0.192
**IL-6** (pg/mL)	20.5 (14.3;29.6)	28.7 (17.2;36.8)	40.5 (11.2;57.8)	0.390
**MKR** **Males**	**Non-Operated** ***n* = 8**	**Cancer** ***n* = 16**	**M–W** **Test**	
**Glucose** (mg/dL)	207.5 (189.3; 237.0)	171.0 (151.3; 199.5)	0.037	
**Insulin** (pg/mL)	10780.8 (8810.5; 12018.1)	8043.4 (5268.8; 10373.4)	0.126	
**C-Peptide** (pg/mL)	5238.0 (4263.1; 5635.3)	2168.2 (1383.7; 3413.6)	0.043	
**Leptin** (pg/mL)	1344.1 (1182.7; 1524.9)	808.2 (640.4; 1077.6)	0.014	
**IL-6** (pg/mL)	17.7 (12.1; 23.6)	73.6 (54.8; 152.7)	0.009	

## Supplementary Materials

Supplementary materials can be found at http://www.mdpi.com/1422-0067/19/4/1198/s1.

## Abbreviations

EAC	Esophageal Adenocarcinoma
ERK	Extracellular signal-Regulated Kinase
ESCC	Esophageal Squamous Cell Carcinoma
GERD	Gastro-Esophageal Reflux Disease
HER2	Human Epidermal growth factor receptor 2
IGF1R	Insulin-like growth factor 1 receptor
IL-6	Interleukin 6
IR	Insulin receptor
MAPK	Mitogen-Activated Protein Kinase
pS	Phosphorylated serine residue
pY	Phosphorylated tyrosine residue
Thr	Threonine residue
Tyr	Tyrosine residue
