# Supplementary material for: Hyperinsulinemia Promotes Esophageal Cancer Development in a Surgically-Induced Duodeno-Esophageal Reflux Murine Model
Source: Int J Mol Sci. 2018 Apr 14;19(4):1198. doi: 10.3390/ijms19041198 (PMC5979452; doi:10.3390/ijms19041198)
Supplement: Supplementary file 1 [file ijms-19-01198-s001.pdf]

Table S1

GROUP	WT F (A)	WT M (B)	MKR F (C)	MKR M (D)	K-W test	A *vs.* B	C *vs.* D	A *vs.* C	B *vs.* D
**GLUCOSE** (mg/dL)	130.0 (114.3;142.3)	158.0 (148.8;181.5)	151.0 (131.5; 159.8)	193.0 (161.8; 206.8)	0.078	0.115	0.046	0.318	0.141
**INSULIN** (pg/mL)	286.3 (273.8;440.3)	812.0 (736.4;924.2)	2523.6 (1445.1;4244.5)	5070.3 (2675.0;13761.5)	$1.3\cdot10^{-5}$	$1.6\cdot10^{-3}$	0.076	$7.8\cdot10^{-4}$	$9.1\cdot10^{-4}$
**C-PEPTIDE** (pg/mL)	391.7 (377.3;432.3)	774.0 (588.5;892.9)	1270.7 (859.2;1952.0)	1534.2 (954.9;2101.7)	$6.1\cdot10^{-4}$	0.027	0.700	$3.3\cdot10^{-3}$	$5.5\cdot10^{-3}$
**LEPTIN** (pg/mL)	2285.7 (2038.7;2904.3)	4400.1 (3664.3;5778.7)	1095.9 (933.7;1204.7)	1089.2 (917.1;1441.4)	$3.6\cdot10^{-5}$	0.021	0.834	$7.8\cdot10^{-4}$	$7.8\cdot10^{-4}$

Table S1. Basal serum metabolic parameters in non-operated WT and MKR mice at 13 weeks of age. Data were expressed as Median (Q1; Q3). K-W test indicates Kruskal-Wallis test result used for multiple comparisons among the four groups of mice. The columns with the header in gray show the Mann-Whitney U-test result used to compare data distribution between two groups of mice. A p-value lower than 0.008 was considered statistically significant (Bonferroni correction).

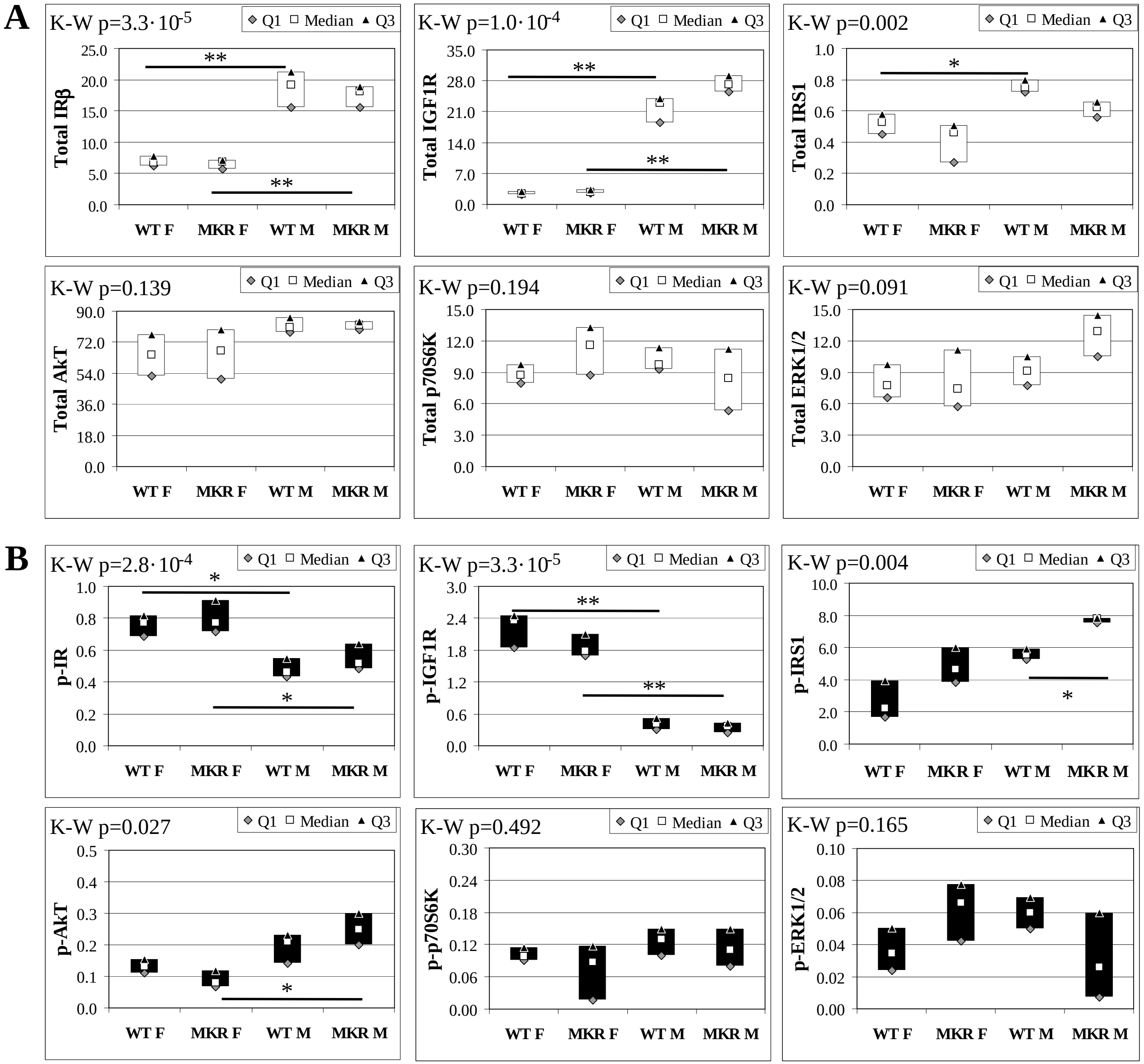
A
K-W p=3.3·10-5
Q1 Median Q3
Total IRβ
25.0
20.0
15.0
10.0
5.0
0.0
**
**
WT F
MKR F
WT M
MKR M
K-W p=1.0·10-4
Total IGF1R
35.0
28.0
21.0
14.0
7.0
0.0
**
**
K-W p=0.002
Total IRS1
1.0
0.8
0.6
0.4
0.2
0.0
*
K-W p=0.139
Total AkT
90.0
72.0
54.0
36.0
18.0
0.0
K-W p=0.194
Total p70S6K
15.0
12.0
9.0
6.0
3.0
0.0
K-W p=0.091
Total ERK1/2
15.0
12.0
9.0
6.0
3.0
0.0
B
K-W p=2.8·10-4
p-IR
1.0
0.8
0.6
0.4
0.2
0.0
*
*
K-W p=3.3·10-5
p-IGF1R
3.0
2.4
1.8
1.2
0.6
0.0
**
**
K-W p=0.004
p-IRS1
10.0
8.0
6.0
4.0
2.0
0.0
*
K-W p=0.027
p-AkT
0.5
0.4
0.3
0.2
0.1
0.0
*
K-W p=0.492
p-p70S6K
0.30
0.24
0.18
0.12
0.06
0.00
K-W p=0.165
p-ERK1/2
0.10
0.08
0.06
0.04
0.02
0.00

Figure S1. Basal insulin signaling in non-operated mice esophageal tissue at 13 weeks of age. Data are represented as Median (Q1; Q3). K-W indicates Kruskal-Wallis test result used for multiple comparisons among the four groups of mice. Mann-Whitney U-test was performed to evaluate the differences between the two groups WT and MKR with the same gender. *p<0.008;**p<8·10-4. **A.** Total protein expression: the quantity of each total protein considered was expressed in ng/100 mg of total protein extracted by the distal mice esophageal segment. **B.** Phosphorylated protein expression: the amount of the six phospho- proteins considered was expressed as phosphorylated Units for each ng of the corresponding total protein amount. Proteins were quantified by Luminex-xMAP Technology on the basis of a standard curve. Beta-tubulin expression was used as internal loading control.
